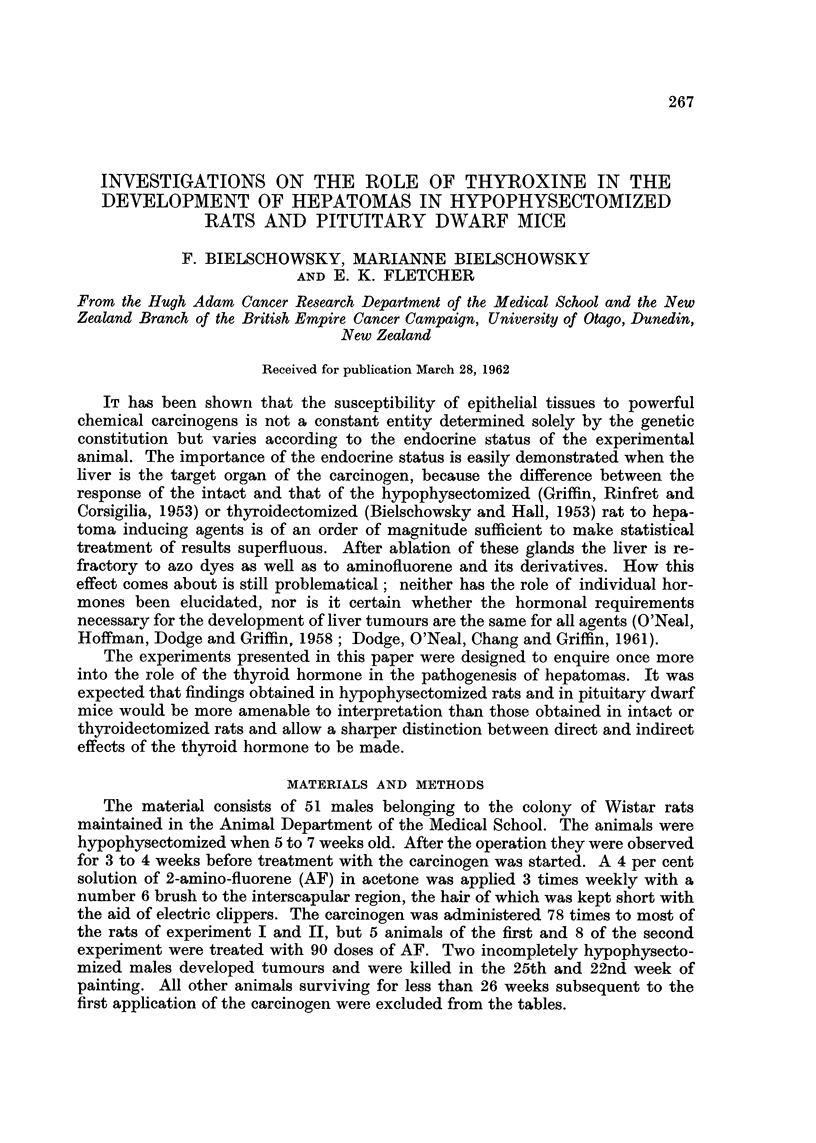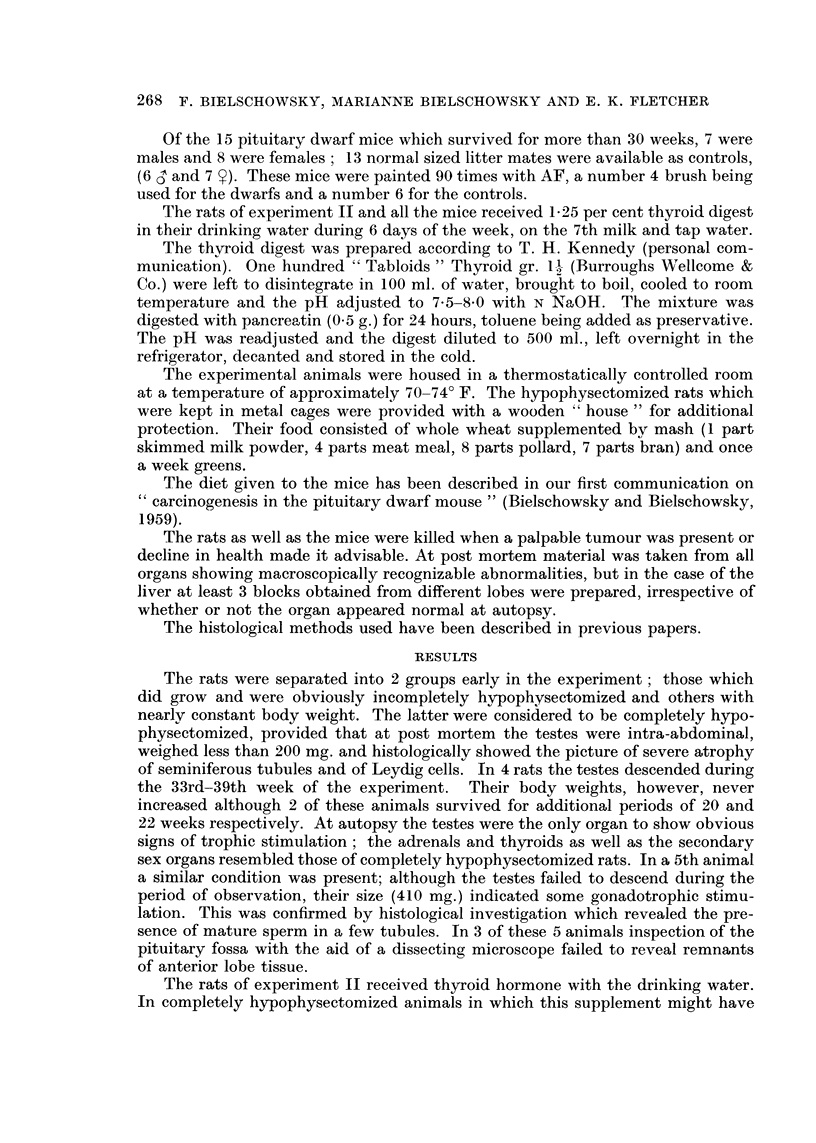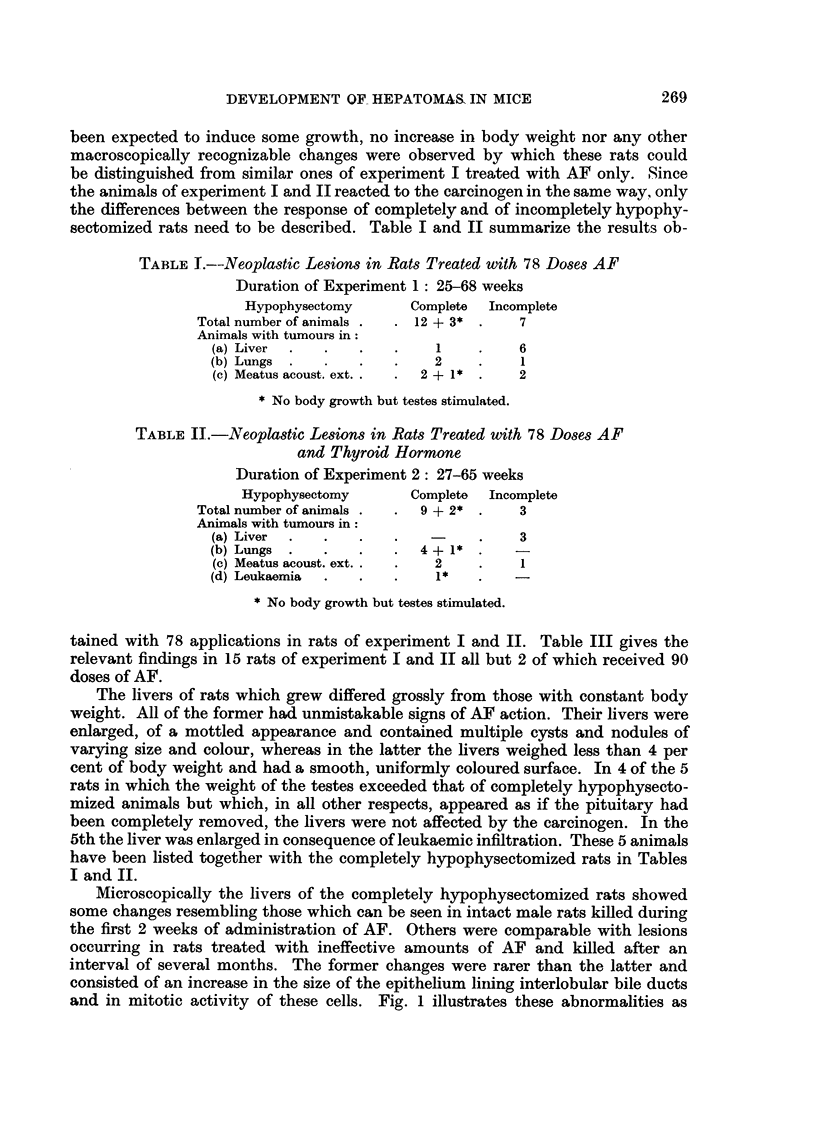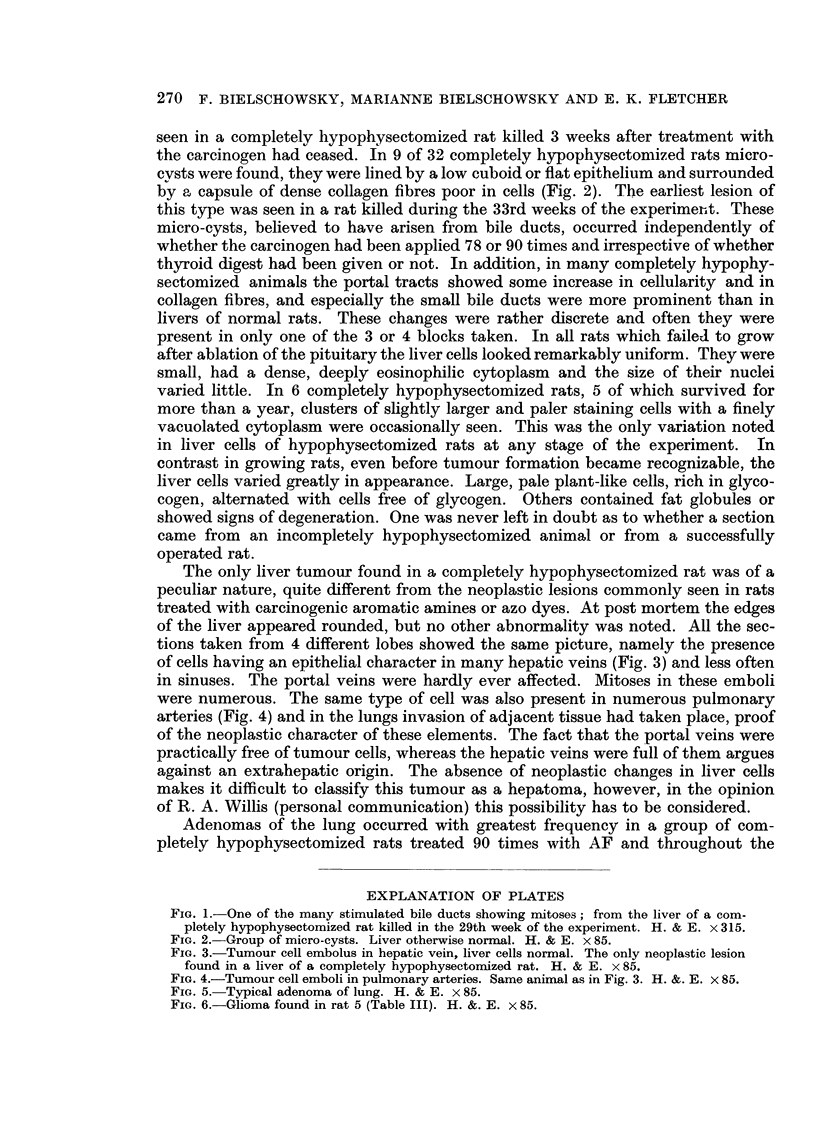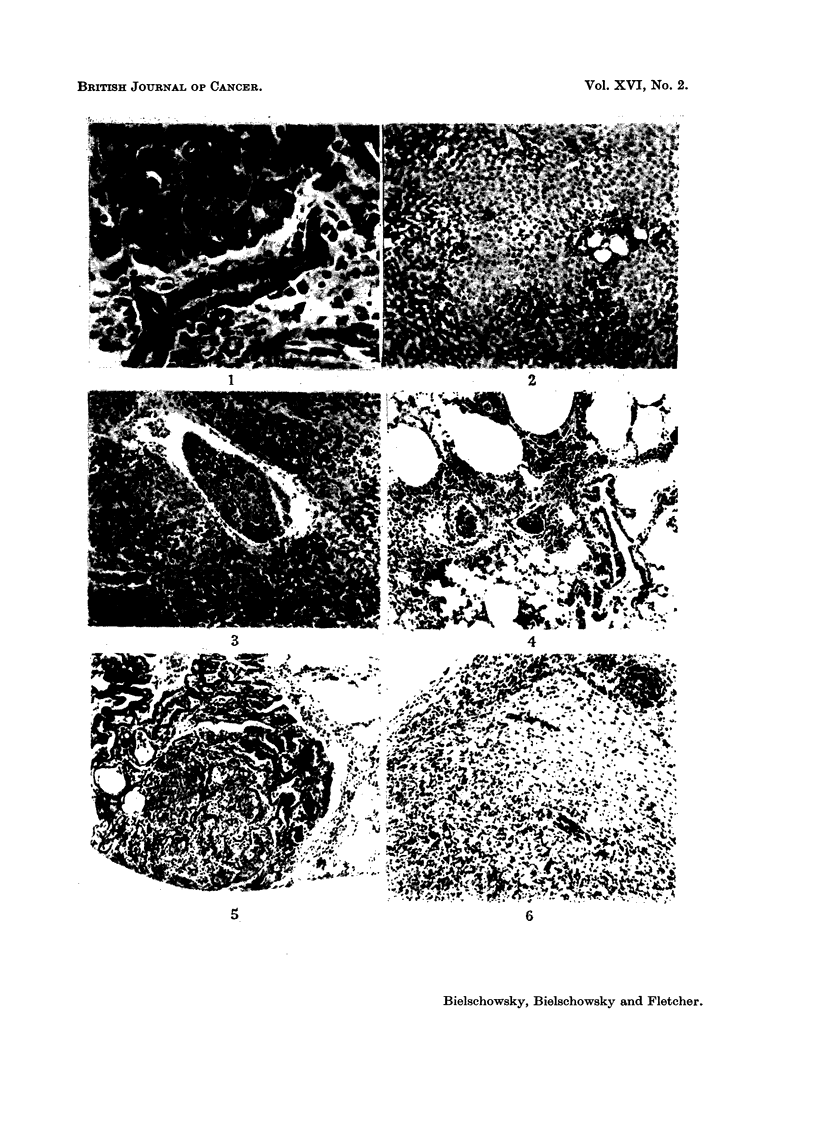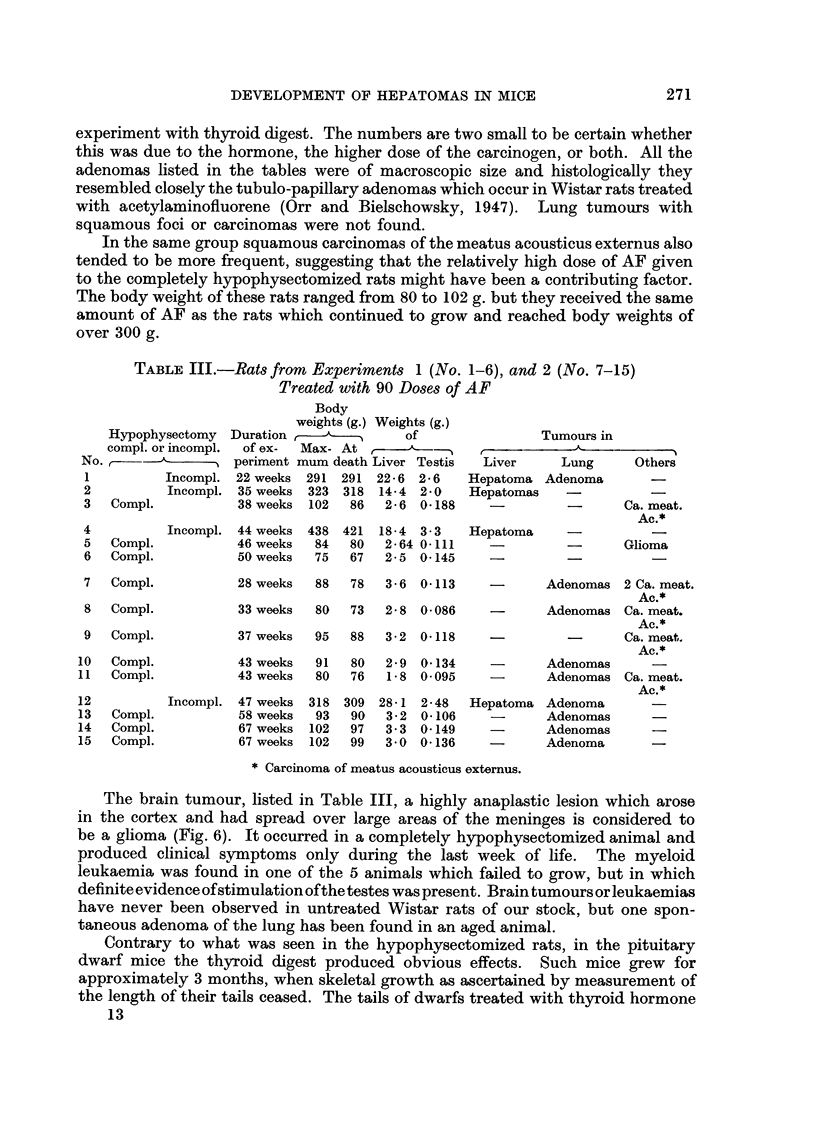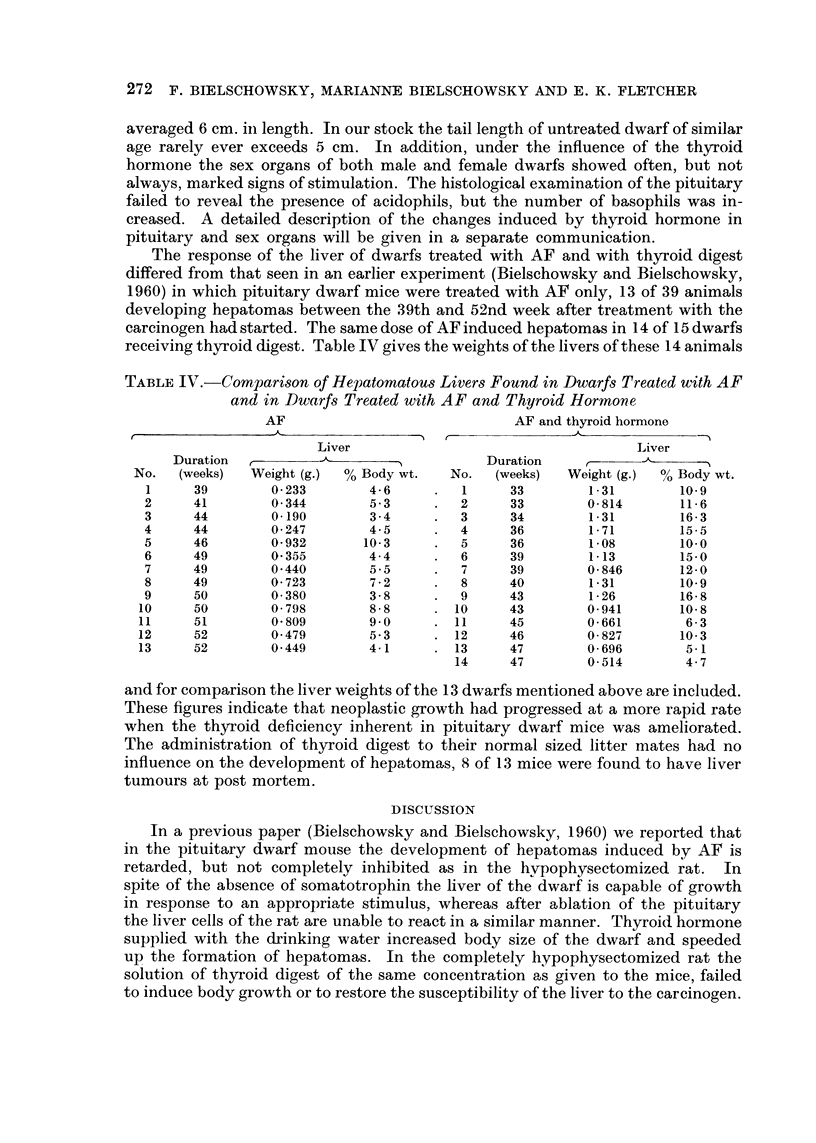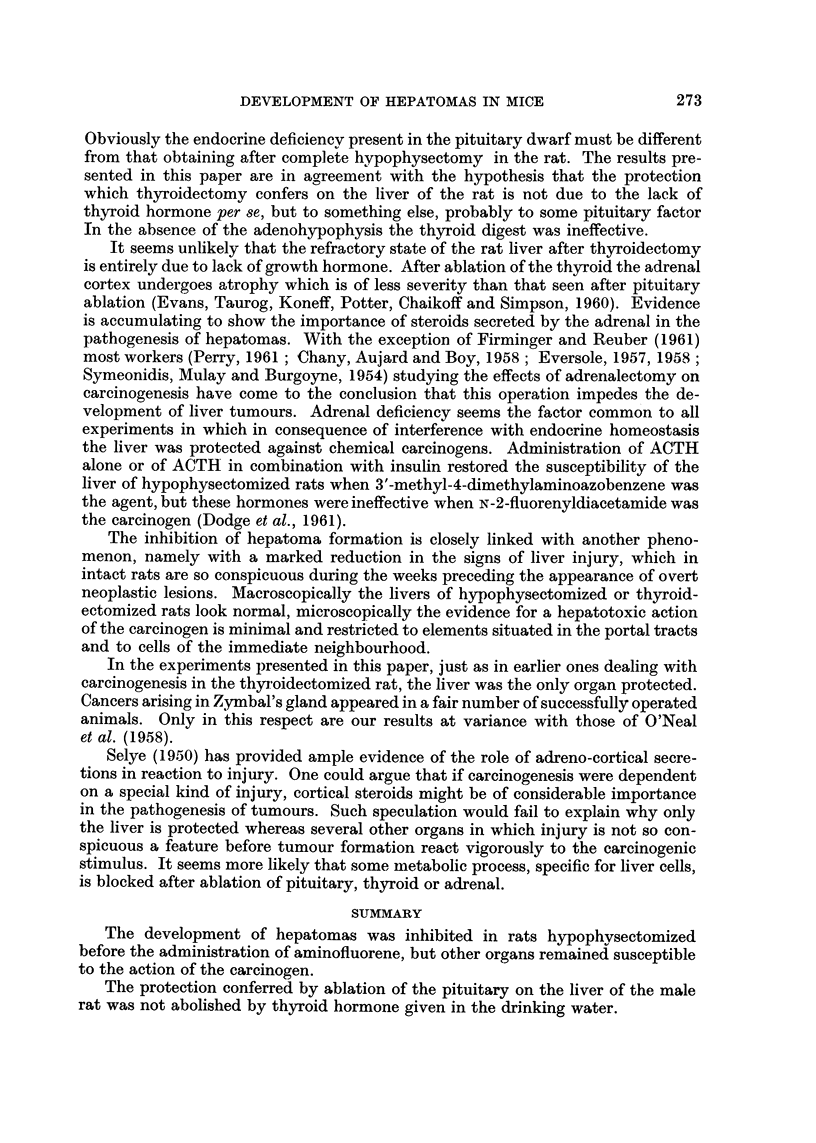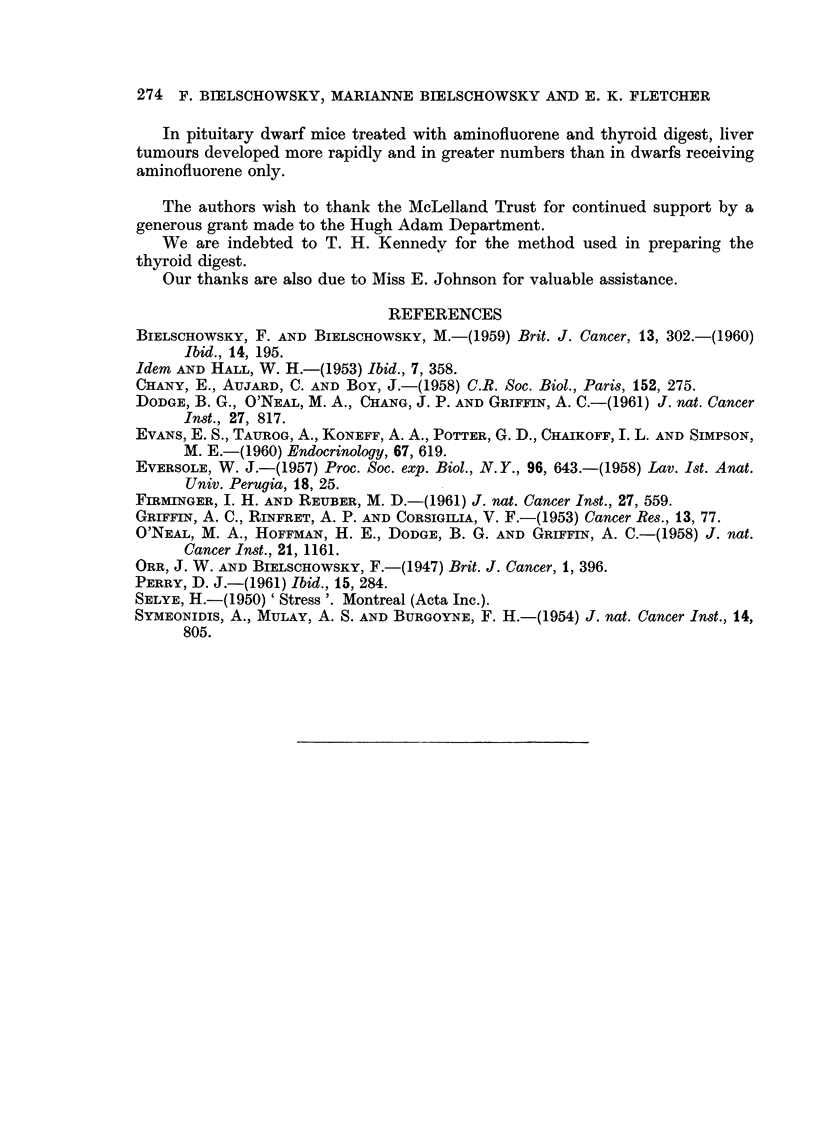# Investigations on the Role of Thyroxine in the Development of Hepatomas in Hypophysectomized Rats and Pituitary Dwarf Mice

**DOI:** 10.1038/bjc.1962.31

**Published:** 1962-06

**Authors:** F. Bielschowsky, Marianne Bielschowsky, E. K. Fletcher

## Abstract

**Images:**


### INVESTIGATIONS ON THE ROLE OF THYROXINE IN THE DEVELOPMENT OF HEPATOMAS IN HYPOPHYSECTOMIZED RATS AND PITUITARY DWARF MICE

F. BIELSCHOWSKY, MARIANNE BIELSCHOWSKY AND E. K. FLETCHER

*From the Hugh Adam Cancer Research Department of the Medical School and the New Zealand Branch of the British Empire Cancer Campaign, University of Otago, Dunedin, New Zealand*

Received for publication March 28, 1962

It has been shown that the susceptibility of epithelial tissues to powerful chemical carcinogens is not a constant entity determined solely by the genetic constitution but varies according to the endocrine status of the experimental animal. The importance of the endocrine status is easily demonstrated when the liver is the target organ of the carcinogen, because the difference between the response of the intact and that of the hypophysectomized (Griffin, Rinfret and Corsigilia, 1953) or thyroidectomized (Bielschowsky and Hall, 1953) rat to hepatoma inducing agents is of an order of magnitude sufficient to make statistical treatment of results superfluous. After ablation of these glands the liver is refractory to azo dyes as well as to aminofluorene and its derivatives. How this effect comes about is still problematical; neither has the role of individual hormones been elucidated, nor is it certain whether the hormonal requirements necessary for the development of liver tumours are the same for all agents (O'Neal, Hoffman, Dodge and Griffin, 1958; Dodge, O'Neal, Chang and Griffin, 1961).

The experiments presented in this paper were designed to enquire once more into the role of the thyroid hormone in the pathogenesis of hepatomas. It was expected that findings obtained in hypophysectomized rats and in pituitary dwarf mice would be more amenable to interpretation than those obtained in intact or thyroidectomized rats and allow a sharper distinction between direct and indirect effects of the thyroid hormone to be made.

### MATERIALS AND METHODS

The material consists of 51 males belonging to the colony of Wistar rats maintained in the Animal Department of the Medical School. The animals were hypophysectomized when 5 to 7 weeks old. After the operation they were observed for 3 to 4 weeks before treatment with the carcinogen was started. A 4 per cent solution of 2-amino-fluorene (AF) in acetone was applied 3 times weekly with a number 6 brush to the interscapular region, the hair of which was kept short with the aid of electric clippers. The carcinogen was administered 78 times to most of the rats of experiment I and II, but 5 animals of the first and 8 of the second experiment were treated with 90 doses of AF. Two incompletely hypophysectomized males developed tumours and were killed in the 25th and 22nd week of painting. All other animals surviving for less than 26 weeks subsequent to the first application of the carcinogen were excluded from the tables.

Of the 15 pituitary dwarf mice which survived for more than 30 weeks, 7 were males and 8 were females ; 13 normal sized litter mates were available as controls, (6 ♂ and 7 ♀). These mice were painted 90 times with AF, a number 4 brush being used for the dwarfs and a number 6 for the controls.

The rats of experiment II and all the mice received 1·25 per cent thyroid digest in their drinking water during 6 days of the week, on the 7th milk and tap water.

The thyroid digest was prepared according to T. H. Kennedy (personal communication). One hundred " Tabloids " Thyroid gr. 1½ (Burroughs Wellcome & Co.) were left to disintegrate in 100 ml. of water, brought to boil, cooled to room temperature and the pH adjusted to 7·5–8·0 with N NaOH. The mixture was digested with pancreatin (0·5 g.) for 24 hours, toluene being added as preservative. The pH was readjusted and the digest diluted to 500 ml., left overnight in the refrigerator, decanted and stored in the cold.

The experimental animals were housed in a thermostatically controlled room at a temperature of approximately 70–74° F. The hypophysectomized rats which were kept in metal cages were provided with a wooden " house " for additional protection. Their food consisted of whole wheat supplemented by mash (1 part skimmed milk powder, 4 parts meat meal, 8 parts pollard, 7 parts bran) and once a week greens.

The diet given to the mice has been described in our first communication on " carcinogenesis in the pituitary dwarf mouse " (Bielschowsky and Bielschowsky, 1959).

The rats as well as the mice were killed when a palpable tumour was present or decline in health made it advisable. At post mortem material was taken from all organs showing macroscopically recognizable abnormalities, but in the case of the liver at least 3 blocks obtained from different lobes were prepared, irrespective of whether or not the organ appeared normal at autopsy.

The histological methods used have been described in previous papers.

### RESULTS

The rats were separated into 2 groups early in the experiment ; those which did grow and were obviously incompletely hypophysectomized and others with nearly constant body weight. The latter were considered to be completely hypophysectomized, provided that at post mortem the testes were intra-abdominal, weighed less than 200 mg. and histologically showed the picture of severe atrophy of seminiferous tubules and of Leydig cells. In 4 rats the testes descended during the 33rd–39th week of the experiment. Their body weights, however, never increased although 2 of these animals survived for additional periods of 20 and 22 weeks respectively. At autopsy the testes were the only organ to show obvious signs of trophic stimulation ; the adrenals and thyroids as well as the secondary sex organs resembled those of completely hypophysectomized rats. In a 5th animal a similar condition was present; although the testes failed to descend during the period of observation, their size (410 mg.) indicated some gonadotrophic stimulation. This was confirmed by histological investigation which revealed the presence of mature sperm in a few tubules. In 3 of these 5 animals inspection of the pituitary fossa with the aid of a dissecting microscope failed to reveal remnants of anterior lobe tissue.

The rats of experiment II received thyroid hormone with the drinking water. In completely hypophysectomized animals in which this supplement might have been expected to induce some growth, no increase in body weight nor any other macroscopically recognizable changes were observed by which these rats could be distinguished from similar ones of experiment I treated with AF only. Since the animals of experiment I and II reacted to the carcinogen in the same way, only the differences between the response of completely and of incompletely hypophysectomized rats need to be described. Table I and II summarize the results obtained with 78 applications in rats of experiment I and II. Table III gives the relevant findings in 15 rats of experiment I and II all but 2 of which received 90 doses of AF.

TABLE I.—*Neoplastic Lesions in Rats Treated with* 78 *Doses AF*

Duration of Experiment 1 : 25–68 weeks

| Hypophysectomy | Complete | Incomplete |
|---|---|---|
| Total number of animals | 12 + 3* | 7 |
| Animals with tumours in : | | |
| (a) Liver | 1 | 6 |
| (b) Lungs | 2 | 1 |
| (c) Meatus acoust. ext. | 2 + 1* | 2 |

* No body growth but testes stimulated.

TABLE II.—*Neoplastic Lesions in Rats Treated with* 78 *Doses AF and Thyroid Hormone*

Duration of Experiment 2 : 27–65 weeks

| Hypophysectomy | Complete | Incomplete |
|---|---|---|
| Total number of animals | 9 + 2* | 3 |
| Animals with tumours in : | | |
| (a) Liver | — | 3 |
| (b) Lungs | 4 + 1* | — |
| (c) Meatus acoust. ext. | 2 | 1 |
| (d) Leukaemia | 1* | — |

* No body growth but testes stimulated.

The livers of rats which grew differed grossly from those with constant body weight. All of the former had unmistakable signs of AF action. Their livers were enlarged, of a mottled appearance and contained multiple cysts and nodules of varying size and colour, whereas in the latter the livers weighed less than 4 per cent of body weight and had a smooth, uniformly coloured surface. In 4 of the 5 rats in which the weight of the testes exceeded that of completely hypophysectomized animals but which, in all other respects, appeared as if the pituitary had been completely removed, the livers were not affected by the carcinogen. In the 5th the liver was enlarged in consequence of leukaemic infiltration. These 5 animals have been listed together with the completely hypophysectomized rats in Tables I and II.

Microscopically the livers of the completely hypophysectomized rats showed some changes resembling those which can be seen in intact male rats killed during the first 2 weeks of administration of AF. Others were comparable with lesions occurring in rats treated with ineffective amounts of AF and killed after an interval of several months. The former changes were rarer than the latter and consisted of an increase in the size of the epithelium lining interlobular bile ducts and in mitotic activity of these cells. Fig. 1 illustrates these abnormalities as seen in a completely hypophysectomized rat killed 3 weeks after treatment with the carcinogen had ceased. In 9 of 32 completely hypophysectomized rats micro-cysts were found, they were lined by a low cuboid or flat epithelium and surrounded by a capsule of dense collagen fibres poor in cells (Fig. 2). The earliest lesion of this type was seen in a rat killed during the 33rd weeks of the experiment. These micro-cysts, believed to have arisen from bile ducts, occurred independently of whether the carcinogen had been applied 78 or 90 times and irrespective of whether thyroid digest had been given or not. In addition, in many completely hypophysectomized animals the portal tracts showed some increase in cellularity and in collagen fibres, and especially the small bile ducts were more prominent than in livers of normal rats. These changes were rather discrete and often they were present in only one of the 3 or 4 blocks taken. In all rats which failed to grow after ablation of the pituitary the liver cells looked remarkably uniform. They were small, had a dense, deeply eosinophilic cytoplasm and the size of their nuclei varied little. In 6 completely hypophysectomized rats, 5 of which survived for more than a year, clusters of slightly larger and paler staining cells with a finely vacuolated cytoplasm were occasionally seen. This was the only variation noted in liver cells of hypophysectomized rats at any stage of the experiment. In contrast in growing rats, even before tumour formation became recognizable, the liver cells varied greatly in appearance. Large, pale plant-like cells, rich in glycogen, alternated with cells free of glycogen. Others contained fat globules or showed signs of degeneration. One was never left in doubt as to whether a section came from an incompletely hypophysectomized animal or from a successfully operated rat.

The only liver tumour found in a completely hypophysectomized rat was of a peculiar nature, quite different from the neoplastic lesions commonly seen in rats treated with carcinogenic aromatic amines or azo dyes. At post mortem the edges of the liver appeared rounded, but no other abnormality was noted. All the sections taken from 4 different lobes showed the same picture, namely the presence of cells having an epithelial character in many hepatic veins (Fig. 3) and less often in sinuses. The portal veins were hardly ever affected. Mitoses in these emboli were numerous. The same type of cell was also present in numerous pulmonary arteries (Fig. 4) and in the lungs invasion of adjacent tissue had taken place, proof of the neoplastic character of these elements. The fact that the portal veins were practically free of tumour cells, whereas the hepatic veins were full of them argues against an extrahepatic origin. The absence of neoplastic changes in liver cells makes it difficult to classify this tumour as a hepatoma, however, in the opinion of R. A. Willis (personal communication) this possibility has to be considered.

Adenomas of the lung occurred with greatest frequency in a group of completely hypophysectomized rats treated 90 times with AF and throughout the

---

EXPLANATION OF PLATES

FIG. 1.—One of the many stimulated bile ducts showing mitoses ; from the liver of a completely hypophysectomized rat killed in the 29th week of the experiment. H. & E. ×315.
FIG. 2.—Group of micro-cysts. Liver otherwise normal. H. & E. ×85.
FIG. 3.—Tumour cell embolus in hepatic vein, liver cells normal. The only neoplastic lesion found in a liver of a completely hypophysectomized rat. H. & E. ×85.
FIG. 4.—Tumour cell emboli in pulmonary arteries. Same animal as in Fig. 3. H. &. E. ×85.
FIG. 5.—Typical adenoma of lung. H. & E. ×85.
FIG. 6.—Glioma found in rat 5 (Table III). H. &. E. ×85.

1

2

3

4

5

6

experiment with thyroid digest. The numbers are two small to be certain whether this was due to the hormone, the higher dose of the carcinogen, or both. All the adenomas listed in the tables were of macroscopic size and histologically they resembled closely the tubulo-papillary adenomas which occur in Wistar rats treated with acetylaminofluorene (Orr and Bielschowsky, 1947). Lung tumours with squamous foci or carcinomas were not found.

In the same group squamous carcinomas of the meatus acousticus externus also tended to be more frequent, suggesting that the relatively high dose of AF given to the completely hypophysectomized rats might have been a contributing factor. The body weight of these rats ranged from 80 to 102 g. but they received the same amount of AF as the rats which continued to grow and reached body weights of over 300 g.

TABLE III.—*Rats from Experiments* 1 (*No.* 1–6), *and* 2 (*No.* 7–15) *Treated with* 90 *Doses of AF*

| No. | Hypophysectomy compl. or incompl. | | Duration of experiment | Body weights (g.) | | Weights (g.) of | | Tumours in | | |
|---|---|---|---|---|---|---|---|---|---|---|
| | | | | Maximum | At death | Liver | Testis | Liver | Lung | Others |
| 1 | | Incompl. | 22 weeks | 291 | 291 | 22·6 | 2·6 | Hepatoma | Adenoma | — |
| 2 | | Incompl. | 35 weeks | 323 | 318 | 14·4 | 2·0 | Hepatomas | — | — |
| 3 | Compl. | | 38 weeks | 102 | 86 | 2·6 | 0·188 | — | — | Ca. meat. Ac.* |
| 4 | | Incompl. | 44 weeks | 438 | 421 | 18·4 | 3·3 | Hepatoma | — | — |
| 5 | Compl. | | 46 weeks | 84 | 80 | 2·64 | 0·111 | — | — | Glioma |
| 6 | Compl. | | 50 weeks | 75 | 67 | 2·5 | 0·145 | — | — | — |
| 7 | Compl. | | 28 weeks | 88 | 78 | 3·6 | 0·113 | — | Adenomas | 2 Ca. meat. Ac.* |
| 8 | Compl. | | 33 weeks | 80 | 73 | 2·8 | 0·086 | — | Adenomas | Ca. meat. Ac.* |
| 9 | Compl. | | 37 weeks | 95 | 88 | 3·2 | 0·118 | — | — | Ca. meat. Ac.* |
| 10 | Compl. | | 43 weeks | 91 | 80 | 2·9 | 0·134 | — | Adenomas | — |
| 11 | Compl. | | 43 weeks | 80 | 76 | 1·8 | 0·095 | — | Adenomas | Ca. meat. Ac.* |
| 12 | | Incompl. | 47 weeks | 318 | 309 | 28·1 | 2·48 | Hepatoma | Adenoma | — |
| 13 | Compl. | | 58 weeks | 93 | 90 | 3·2 | 0·106 | — | Adenomas | — |
| 14 | Compl. | | 67 weeks | 102 | 97 | 3·3 | 0·149 | — | Adenomas | — |
| 15 | Compl. | | 67 weeks | 102 | 99 | 3·0 | 0·136 | — | Adenoma | — |

* Carcinoma of meatus acousticus externus.

The brain tumour, listed in Table III, a highly anaplastic lesion which arose in the cortex and had spread over large areas of the meninges is considered to be a glioma (Fig. 6). It occurred in a completely hypophysectomized animal and produced clinical symptoms only during the last week of life. The myeloid leukaemia was found in one of the 5 animals which failed to grow, but in which definite evidence of stimulation of the testes was present. Brain tumours or leukaemias have never been observed in untreated Wistar rats of our stock, but one spontaneous adenoma of the lung has been found in an aged animal.

Contrary to what was seen in the hypophysectomized rats, in the pituitary dwarf mice the thyroid digest produced obvious effects. Such mice grew for approximately 3 months, when skeletal growth as ascertained by measurement of the length of their tails ceased. The tails of dwarfs treated with thyroid hormone averaged 6 cm. in length. In our stock the tail length of untreated dwarf of similar age rarely ever exceeds 5 cm. In addition, under the influence of the thyroid hormone the sex organs of both male and female dwarfs showed often, but not always, marked signs of stimulation. The histological examination of the pituitary failed to reveal the presence of acidophils, but the number of basophils was increased. A detailed description of the changes induced by thyroid hormone in pituitary and sex organs will be given in a separate communication.

The response of the liver of dwarfs treated with AF and with thyroid digest differed from that seen in an earlier experiment (Bielschowsky and Bielschowsky, 1960) in which pituitary dwarf mice were treated with AF only, 13 of 39 animals developing hepatomas between the 39th and 52nd week after treatment with the carcinogen had started. The same dose of AF induced hepatomas in 14 of 15 dwarfs receiving thyroid digest. Table IV gives the weights of the livers of these 14 animals

TABLE IV.—*Comparison of Hepatomatous Livers Found in Dwarfs Treated with AF and in Dwarfs Treated with AF and Thyroid Hormone*

| AF | | | | AF and thyroid hormone | | | |
|---|---|---|---|---|---|---|---|
| | | Liver | | | | Liver | |
| No. | Duration (weeks) | Weight (g.) | % Body wt. | No. | Duration (weeks) | Weight (g.) | % Body wt. |
| 1 | 39 | 0·233 | 4·6 | 1 | 33 | 1·31 | 10·9 |
| 2 | 41 | 0·344 | 5·3 | 2 | 33 | 0·814 | 11·6 |
| 3 | 44 | 0·190 | 3·4 | 3 | 34 | 1·31 | 16·3 |
| 4 | 44 | 0·247 | 4·5 | 4 | 36 | 1·71 | 15·5 |
| 5 | 46 | 0·932 | 10·3 | 5 | 36 | 1·08 | 10·0 |
| 6 | 49 | 0·355 | 4·4 | 6 | 39 | 1·13 | 15·0 |
| 7 | 49 | 0·440 | 5·5 | 7 | 39 | 0·846 | 12·0 |
| 8 | 49 | 0·723 | 7·2 | 8 | 40 | 1·31 | 10·9 |
| 9 | 50 | 0·380 | 3·8 | 9 | 43 | 1·26 | 16·8 |
| 10 | 50 | 0·798 | 8·8 | 10 | 43 | 0·941 | 10·8 |
| 11 | 51 | 0·809 | 9·0 | 11 | 45 | 0·661 | 6·3 |
| 12 | 52 | 0·479 | 5·3 | 12 | 46 | 0·827 | 10·3 |
| 13 | 52 | 0·449 | 4·1 | 13 | 47 | 0·696 | 5·1 |
| | | | | 14 | 47 | 0·514 | 4·7 |

and for comparison the liver weights of the 13 dwarfs mentioned above are included. These figures indicate that neoplastic growth had progressed at a more rapid rate when the thyroid deficiency inherent in pituitary dwarf mice was ameliorated. The administration of thyroid digest to their normal sized litter mates had no influence on the development of hepatomas, 8 of 13 mice were found to have liver tumours at post mortem.

### DISCUSSION

In a previous paper (Bielschowsky and Bielschowsky, 1960) we reported that in the pituitary dwarf mouse the development of hepatomas induced by AF is retarded, but not completely inhibited as in the hypophysectomized rat. In spite of the absence of somatotrophin the liver of the dwarf is capable of growth in response to an appropriate stimulus, whereas after ablation of the pituitary the liver cells of the rat are unable to react in a similar manner. Thyroid hormone supplied with the drinking water increased body size of the dwarf and speeded up the formation of hepatomas. In the completely hypophysectomized rat the solution of thyroid digest of the same concentration as given to the mice, failed to induce body growth or to restore the susceptibility of the liver to the carcinogen.

Obviously the endocrine deficiency present in the pituitary dwarf must be different from that obtaining after complete hypophysectomy in the rat. The results presented in this paper are in agreement with the hypothesis that the protection which thyroidectomy confers on the liver of the rat is not due to the lack of thyroid hormone *per se*, but to something else, probably to some pituitary factor In the absence of the adenohypophysis the thyroid digest was ineffective.

It seems unlikely that the refractory state of the rat liver after thyroidectomy is entirely due to lack of growth hormone. After ablation of the thyroid the adrenal cortex undergoes atrophy which is of less severity than that seen after pituitary ablation (Evans, Taurog, Koneff, Potter, Chaikoff and Simpson, 1960). Evidence is accumulating to show the importance of steroids secreted by the adrenal in the pathogenesis of hepatomas. With the exception of Firminger and Reuber (1961) most workers (Perry, 1961 ; Chany, Aujard and Boy, 1958 ; Eversole, 1957, 1958 ; Symeonidis, Mulay and Burgoyne, 1954) studying the effects of adrenalectomy on carcinogenesis have come to the conclusion that this operation impedes the development of liver tumours. Adrenal deficiency seems the factor common to all experiments in which in consequence of interference with endocrine homeostasis the liver was protected against chemical carcinogens. Administration of ACTH alone or of ACTH in combination with insulin restored the susceptibility of the liver of hypophysectomized rats when 3′-methyl-4-dimethylaminoazobenzene was the agent, but these hormones were ineffective when N-2-fluorenyldiacetamide was the carcinogen (Dodge *et al.*, 1961).

The inhibition of hepatoma formation is closely linked with another phenomenon, namely with a marked reduction in the signs of liver injury, which in intact rats are so conspicuous during the weeks preceding the appearance of overt neoplastic lesions. Macroscopically the livers of hypophysectomized or thyroidectomized rats look normal, microscopically the evidence for a hepatotoxic action of the carcinogen is minimal and restricted to elements situated in the portal tracts and to cells of the immediate neighbourhood.

In the experiments presented in this paper, just as in earlier ones dealing with carcinogenesis in the thyroidectomized rat, the liver was the only organ protected. Cancers arising in Zymbal's gland appeared in a fair number of successfully operated animals. Only in this respect are our results at variance with those of O'Neal *et al.* (1958).

Selye (1950) has provided ample evidence of the role of adreno-cortical secretions in reaction to injury. One could argue that if carcinogenesis were dependent on a special kind of injury, cortical steroids might be of considerable importance in the pathogenesis of tumours. Such speculation would fail to explain why only the liver is protected whereas several other organs in which injury is not so conspicuous a feature before tumour formation react vigorously to the carcinogenic stimulus. It seems more likely that some metabolic process, specific for liver cells, is blocked after ablation of pituitary, thyroid or adrenal.

### SUMMARY

The development of hepatomas was inhibited in rats hypophysectomized before the administration of aminofluorene, but other organs remained susceptible to the action of the carcinogen.

The protection conferred by ablation of the pituitary on the liver of the male rat was not abolished by thyroid hormone given in the drinking water.

In pituitary dwarf mice treated with aminofluorene and thyroid digest, liver tumours developed more rapidly and in greater numbers than in dwarfs receiving aminofluorene only.

The authors wish to thank the McLelland Trust for continued support by a generous grant made to the Hugh Adam Department.

We are indebted to T. H. Kennedy for the method used in preparing the thyroid digest.

Our thanks are also due to Miss E. Johnson for valuable assistance.

### REFERENCES

BIELSCHOWSKY, F. AND BIELSCHOWSKY, M.—(1959) *Brit. J. Cancer*, **13**, 302.—(1960) *Ibid.*, **14**, 195.

*Idem* AND HALL, W. H.—(1953) *Ibid.*, **7**, 358.

CHANY, E., AUJARD, C. AND BOY, J.—(1958) *C.R. Soc. Biol., Paris*, **152**, 275.

DODGE, B. G., O'NEAL, M. A., CHANG, J. P. AND GRIFFIN, A. C.—(1961) *J. nat. Cancer Inst.*, **27**, 817.

EVANS, E. S., TAUROG, A., KONEFF, A. A., POTTER, G. D., CHAIKOFF, I. L. AND SIMPSON, M. E.—(1960) *Endocrinology*, **67**, 619.

EVERSOLE, W. J.—(1957) *Proc. Soc. exp. Biol., N.Y.*, **96**, 643.—(1958) *Lav. Ist. Anat. Univ. Perugia*, **18**, 25.

FIRMINGER, I. H. AND REUBER, M. D.—(1961) *J. nat. Cancer Inst.*, **27**, 559.

GRIFFIN, A. C., RINFRET, A. P. AND CORSIGILIA, V. F.—(1953) *Cancer Res.*, **13**, 77.

O'NEAL, M. A., HOFFMAN, H. E., DODGE, B. G. AND GRIFFIN, A. C.—(1958) *J. nat. Cancer Inst.*, **21**, 1161.

ORR, J. W. AND BIELSCHOWSKY, F.—(1947) *Brit. J. Cancer*, **1**, 396.

PERRY, D. J.—(1961) *Ibid.*, **15**, 284.

SELYE, H.—(1950) ' Stress '. Montreal (Acta Inc.).

SYMEONIDIS, A., MULAY, A. S. AND BURGOYNE, F. H.—(1954) *J. nat. Cancer Inst.*, **14**, 805.